# Integrin-Linked Kinase Overexpression and Its Oncogenic Role in Promoting Tumorigenicity of Hepatocellular Carcinoma

**DOI:** 10.1371/journal.pone.0016984

**Published:** 2011-02-09

**Authors:** Jenny Chan, Frankie Chi Fat Ko, Yin-Shan Yeung, Irene Oi-Lin Ng, Judy Wai Ping Yam

**Affiliations:** 1 Department of Pathology, Li Ka Shing Faculty of Medicine, The University of Hong Kong, Hong Kong, China; 2 Centre for Cancer Research, Li Ka Shing Faculty of Medicine, The University of Hong Kong, Hong Kong, China; 3 State Key Laboratory for Liver Research, Li Ka Shing Faculty of Medicine, The University of Hong Kong, Hong Kong, China; 4 Liver Cancer and Hepatitis Research Laboratory, Li Ka Shing Faculty of Medicine, The University of Hong Kong, Hong Kong, China; Yonsei University College of Medicine, Republic of Korea

## Abstract

**Background:**

Integrin-linked kinase (ILK) was first discovered as an integrin β1-subunit binding protein. It localizes at the focal adhesions and is involved in cytoskeleton remodeling. ILK overexpression and its dysregulated signaling cascades have been reported in many human cancers. Aberrant expression of ILK influenced a wide range of signaling pathways and cellular functions. Although ILK has been well characterized in many malignancies, its role in hepatocellular carcinoma (HCC) is still largely unknown.

**Methodology/Principal Findings:**

Quantitative PCR analysis was used to examine ILK mRNA expression in HCC clinical samples. It was shown that ILK was overexpressed in 36.9% (21/57) of HCC tissues when compared to the corresponding non-tumorous livers. The overall ILK expression level was significantly higher in tumorous tissues (*P* = 0.004), with a significant stepwise increase in expression level along tumor progression from tumor stage I to IV (*P* = 0.045). ILK knockdown stable clones were established in two HCC cell lines, BEL7402 and HLE, and were subjected to different functional assays. Knockdown of ILK significantly suppressed HCC cell growth, motility and invasion *in vitro* and inhibited tumorigenicity *in vivo*. Western blot analysis revealed a reduced phosphorylated-Akt (pAkt) at Serine-473 expression in ILK knockdown stable clones when compared to control clones.

**Conclusion/Significance:**

This study provides evidence about the clinical relevance of ILK in hepatocarcinogenesis. ILK was found to be progressively elevated along HCC progression. Here our findings also provide the first validation about the oncogenic capacity of ILK *in vivo* by suppressing its expression in HCC cells. The oncogenic role of ILK is implicated to be mediated by Akt pathway.

## Introduction

HCC is the fifth most common cancer worldwide and highly prevalent in southeastern Asia [1]. It displays late diagnosis, poor prognosis and high mortality rate. There is currently no effective treatment for HCC. Only approximately 15% of patients are eligible for tumor resection or liver transplantation, where half of them will experience tumor recurrence 3 years after therapy [2], [3]. HCC is a highly complicated and heterogeneous tumor which results from the aberrant activation of numerous important signaling pathways. Understanding the molecular mechanisms of HCC is of the utmost importance to the search for curative therapy.

ILK was first discovered in 1996 by Hannigan et al. in a yeast-two hybrid experiment screening for integrin β1-subunit interactor [4]. Since then, a large number of studies have been conducted on ILK, attempting to understand the expression and functional roles of ILK in the cells. The ILK gene is located at human chromosome 11, band 11p15.4/15.5. Database searches found that only one ILK gene exists [5]. The ILK protein consists of 452 amino acids and is made up of three major domains – the N-terminal ankyrin repeats, middle pleckstrin homology (PH) domain and C-terminal kinase domain. The major role of ILK is to act as an adaptor protein which allows various intracellular proteins to interact directly with its three domains [6]. The kinase domain is shown to phosphorylate key signaling players such as Akt and GSK3β [7]. ILK has been well characterized to be an important player in the focal adhesions. For example, it physically interacts with cytoplasmic proteins PINCH and parvins to form the PINCH-ILK-parvin (PIP) complex, which helps to translocate ILK to the focal adhesions upon activation by the ECM-stimulated integrin receptors [8], [9]. ILK is also involved in regulating actin polymerization along with its well-characterized scaffolding role in the focal adhesions.

ILK expression and its oncogenic potentials have been studied in various malignancies. Immunohistochemistry (IHC) revealed a higher ILK expression in primary prostate cancer with respect to the adjacent benign prostate hyperplasia. Its expression also positively correlated with tumor grade while inversely correlated with the 5-year survival rate [10]. Similarly, IHC on 53 ovarian cancer samples showed a 100% positive signal, while no stain was observed in normal ovarian epithelium. The staining intensity was observed to increase with tumor grade [11]. ILK has also been shown to have implication in colon cancer progression that higher ILK expression was detected in metastatic tumor and was increased with tumor stage, grade and invasiveness [12]. All these compelling evidences have demonstrated the oncogenic effect of ILK in cancer development. In addition, ILK was reported to be overexpressed in HCC and cirrhosis [13], [14]. Nevertheless, functional characterization of ILK in HCC is still lacking. In this study, we elucidated the role of ILK in hepatocarcinogenesis by assessing its expression in human HCC tumor samples and functionally characterizing its role in HCC cell models. We found that ILK was indeed overexpressed in HCC and exerted oncogenic effect on HCC cell lines both in *vitro* and *in vivo*.

## Results

### ILK overexpression was detected in HCC and correlated significantly with HCC tumor grade

**Figure 1:**
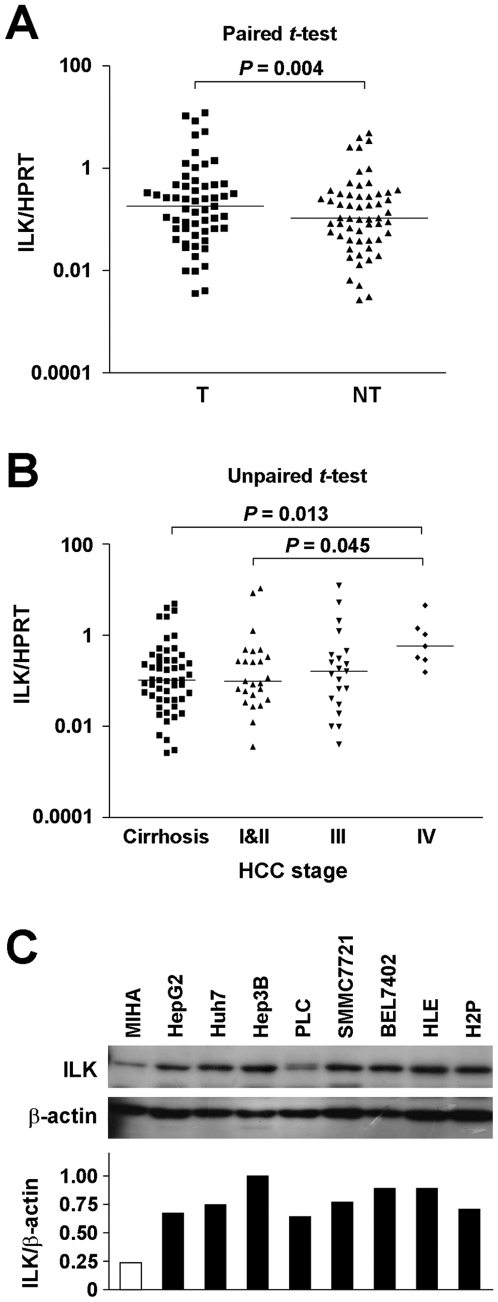
ILK expression was elevated in HCC clinical samples and cell lines. (**A**) ILK mRNA expression was examined by qPCR. ILK was overexpressed in 36.9% of the HCC samples. Cases with T/NT ratio >2 were classified as overexpression, while those with T/NT ratio <0.5 were regarded as underexpression. The remaining cases with 0.5< ratio <2 were regarded as no change in ILK expression. Higher ILK expression (*P* = 0.004) was observed in tumor samples when compared to their non-tumor counterparts. (**B**) A stepwise increase of ILK expression along HCC tumor stage was observed. (**C**) ILK expression in a panel of HCC cell lines was analyzed by western blot analysis. MIHA is an immortalized non-tumorigenic liver cell line. Relative ILK expression normalized with corresponding β-actin expression was shown below.

Quantitative PCR was used to examine the transcript level of ILK in 57 paired HCC tumor samples, each of which consisted of the tumor (T) tissue and the adjacent non-tumorous (NT) liver. Cases with T/NT ratio greater than 2 were classified as overexpression. ILK was found to be overexpressed in 36.9% (21/57) of the cases. When compared the overall ILK expression levels, ILK transcript level was significantly higher in tumor tissues than in non-tumorous liver (*P* = 0.004, unpaired *t*-test) (**Fig. 1A**). HCC tumor stage was classified using the Tumor-Node-Metastasis (pTNM) staging system. Stage I and II were regarded as early stage while stage III and IV were advanced stage. There was no significant difference in ILK expression level between cirrhotic liver and stage I tumor. However, **Fig. 1B** revealed a stepwise increase in ILK expression across tumor stages, namely stage I & II, stage III and stage IV. Notably, ILK expression level was significantly higher in stage IV when compared with cirrhotic liver and early stage HCC (*P* = 0.013 and *P* = 0.045, respectively). This result suggests ILK expression was progressively increases during HCC progression.

### Knockdown of ILK suppressed HCC cell growth

**Figure 2:**
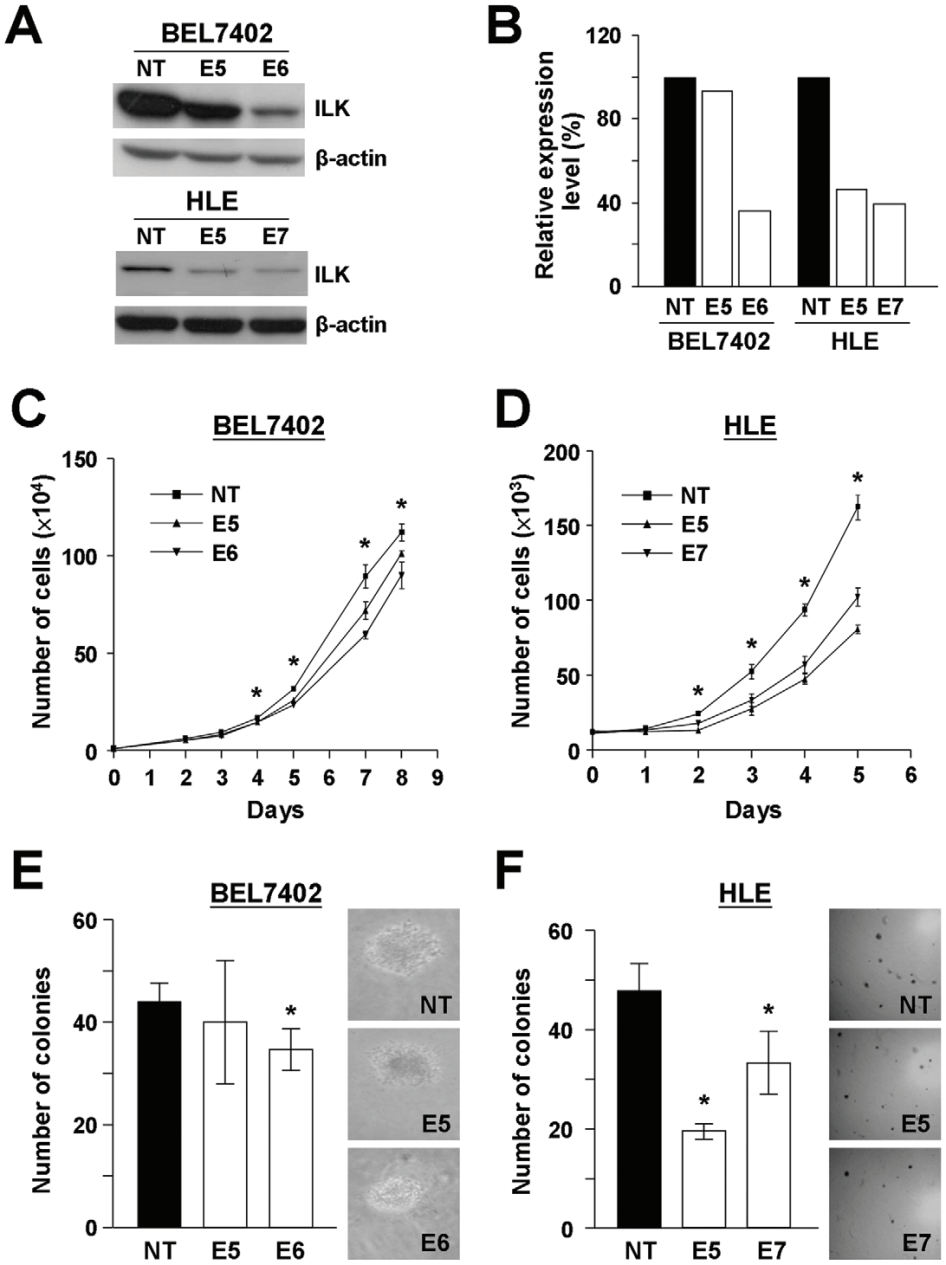
Knockdown of ILK in HCC cell lines impaired HCC cell proliferation and anchorage independent growth. (**A**) One sh-non-target control clone (NT) and two ILK knockdown stable clones were established in BEL7402 (E5, E6) and HLE (E5, E7). (**B**) shILK sequence E6 showed a higher knockdown efficiency than E5 in BEL7402, while both E5 and E7 showed similar knockdown effect in HLE. (**C and D**) Numbers of cells in BEL7402 and HLE ILK knockdown stable clones were counted in triplicates for 5 to 8 consecutive days. Growth curves were plotted to reveal the growth pattern of each cell line. (**E**) Cells from BEL7402 ILK knockdown stable clones were grown in soft agar for 1 month. Colonies were formed from single cells and the number of colonies with diameter >60 µm were counted under microscope. (**F**) Colonies formed from HLE ILK knockdown stable clones were also counted and plotted. Representative pictures of colonies formed from each knockdown stable clone were shown. Asterisk (*) indicates a *P*-value <0.05 with significant difference between NT control and ILK knockdown clones (E5, E6 and E7).

One effective way to understand the physiological role of ILK in HCC was to inhibit the expression of endogenous ILK in HCC cell line models. Western blot analysis of ILK expression in a panel of HCC cell lines revealed that ILK expression in all HCC cell lines was higher than that in MIHA, an immortalized non-tumorigenic liver cell line (**Fig. 1C**). Efficient knockdown of ILK expression was achieved by the successful delivery and expression of short-hairpin (sh) RNA targeting ILK in the cells. In our study, a lentiviral-based delivery system was used to introduce and express two specific ILK shRNA targeting sequences in two HCC cell lines, namely BEL7402 and HLE. Two ILK knockdown stable clones E5 and E6, as well as one non-targeted knockdown control NT were established in BEL7402 (**Fig. 2A**). With reference to NT, a higher ILK knockdown efficiency was achieved in E6 than in E5. Compared with the ILK expression level in NT, 6.8% and 64.2% ILK suppression were observed in E5 and E6, respectively (**Fig. 2B**). Similarly, three knockdown stable clones, NT, E5 and E7 were established in HLE (**Fig. 2A**). Both E5 and E7 clones displayed about 60% knockdown efficiency compared to the control (**Fig. 2B**).

To determine the effect of ILK expression on HCC cell growth, proliferation curves of the ILK knockdown stable clones and the corresponding non-targeted control were determined and compared. As shown in **Fig. 2C**, similar proliferation rates were observed for BEL7402 NT, E5 and E6 during the first 3 days. However, starting from day 4, a significant difference in cell growth was observed between the two ILK knockdown clones and the non-targeted control. NT was found to have a higher proliferation rate when compared to E5 and E6, and this trend persisted till the last day of the proliferation assay. Similar result was observed in HLE ILK knockdown stable clones (**Fig. 2D**). Both ILK knockdown stable clones, E5 and E7, showed a decrease in proliferation when compared to NT starting from day 2. It was also noted that the ILK knockdown efficiency correlated well with the growth suppression effect in both HCC cell lines. Stable clones with better ILK knockdown effect displayed a lower proliferation rate. Results here suggested that ILK knockdown suppressed cell proliferation of HCC cells.

Soft agar colony formation assay was used to assess the ILK knockdown effect on the anchorage independent growth of HCC cells. It was found that single cells seeded were able to form colonies in all three E5, E6 and NT stable clones established in BEL7402. Nevertheless, the size of the colonies varied among the three stable clones. Largest colonies were observed in NT control clone while colonies found in E5 were in moderate size and those in E6 were smallest (**Fig. 2E**). To compare the number of colonies (with diameter greater than 60 µm) formed, number of colonies formed in NT control clone was significantly higher than E6 knockdown clone (*P* = 0.041). HLE ILK knockdown stable clones were also subjected to soft agar colony formation assay. Contrary to BEL7402 cells, not all single cells seeded formed colonies in HLE ILK knockdown stable clones (**Fig. 2F**). The colony number formed in NT was significantly larger than number of colonies formed in E5 (*P* = 0.001) and E7 (*P* = 0.044). Consistent results observed in both BEL7402 and HLE suggested the suppression of anchorage independent growth on HCC cell lines upon ILK knockdown.

### Knockdown of ILK reduced the migratory and invasive potentials of HCC cells

**Figure 3:**
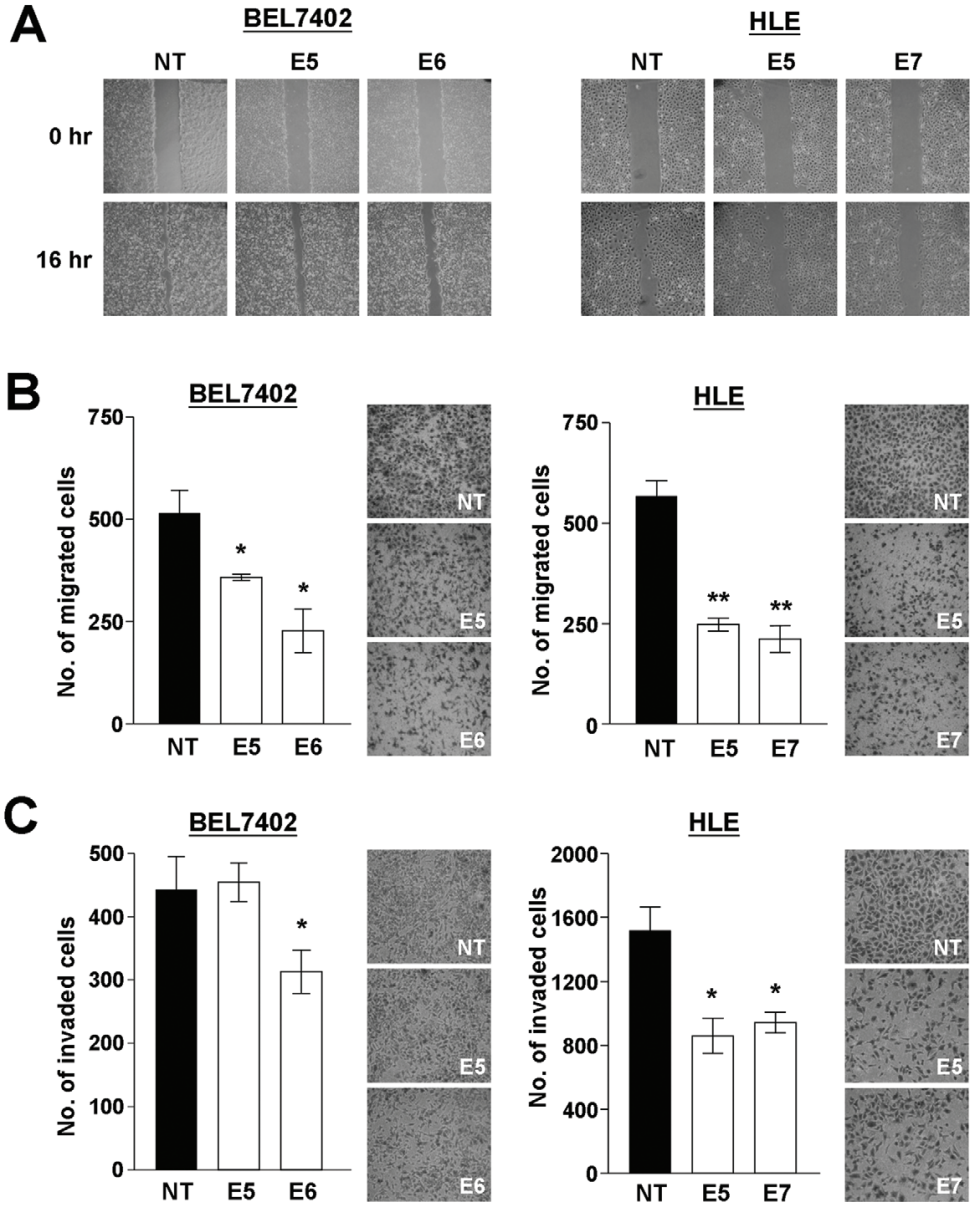
ILK knockdown inhibited cell migration and invasion. (**A**) Cells from BEL7402 and HLE ILK knockdown stable clones were grown to confluence and a wound was created. Closure of the wound was monitored and captured 16 hours after the wound was made. (**B**) BEL7402 and HLE ILK knockdown stable clones were seeded onto migration chambers in triplicate and were allowed to migrate for 24 hours. Cells migrated through the membrane were fixed and visualized by crystal violet staining. (**C**) ILK knockdown stable clones were seeded onto matrigel-coated invasion chamber. BEL7402 was allowed to migrate for 72 hours while HLE required only 24 hours to invade. Cells were fixed, stained and scored. *P<0.05 and **P<0.001 were regarded as statistically significant.

To assess the migratory ability of ILK knockdown stable clones, wound-healing and transwell migration chamber were employed. For wound-healing assay of BEL7402 ILK knockdown clones, the wound in NT control clone was almost closed in 72 hours, yet the wounds in E5 and E6 stable clones were still clearly seen (**Fig. 3A**). Differences in wound-closure ability were even more obvious in HLE ILK knockdown stable clones, in which a difference could be observed 16 hours after the creation of the wound. Larger wound was observed in E5 and E7 stable clones when compared with NT control clone (**Fig. 3A**). Consistently, the transwell migration assay revealed that numbers of migrated cells in BEL7402 NT control clones were significantly higher when compared to E5 (*P* = 0.009) and E6 (*P* = 0.003) stable clones (**Fig. 3B**). Also, both E5 (*P*<0.001) and E7 (*P*<0.001) stable clones of HLE displayed a significant reduction in migratory potential when compared with NT control clones.

Cell invasiveness of ILK knockdown stable clones was assessed by seeding the cells onto matrigel-coated invasion chamber. For BEL7402, more invaded cells were observed in NT control and E5 stable clones, while significantly fewer cells were able to invade through the matrigel in E6 stable clones (*P* = 0.024) (**Fig. 3C**). Results of invasion assay performed using HLE ILK knockdown clones showed a similar outcome. More invaded cells were observed in NT control clone and significantly fewer cells in E5 (*P* = 0.004) and E7 (*P* = 0.004) stable clones (**Fig. 3C**). All these suggested the involvement of ILK in regulating HCC cell motility and invasiveness and knockdown of it suppressed cell invasion.

### Knockdown of ILK impaired HCC cell in vivo tumorigenicity

**Figure 4:**
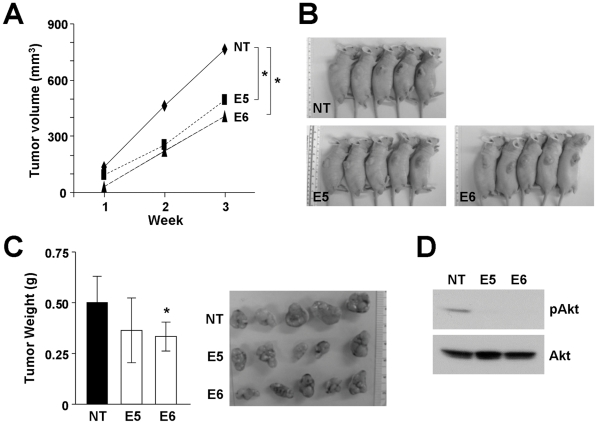
ILK knockdown suppressed *in vivo* tumorigenicity in BEL7402. (**A**) ILK knockdown clones were injected subcutaneously into the right flank of nude mice with 5×10^6^ cells per site. Volumes of the tumors were measured every week by the formula: *V = ½×a (length) x b^2^ (width)*. (**B**) Mice were sacrificed at the third week after subcutaneous injection. (**C**) Tumors were harvested, photographed and weighed. (**D**) Protein was extracted from the excised tumors and analyzed for pAkt expression by western blotting. *P<0.05 was regarded as statistically significant.

Since HLE was unable to form tumor upon subcutaneous injection, only BEL7402 ILK knockdown stable clones were subjected to nude mice injection. After subcutaneous injection of the cells into nude mice, tumor growth was monitored carefully and the size of the tumor formed was measured weekly. Staring from week 2, larger tumors were formed from mice injected with NT control clone, while smaller tumors were formed from E5 and E6 stable clone (**Fig. 4A**). At week three, the animals were sacrificed due to oversize of the tumors (**Fig. 4B**). Tumors were then harvested from the mice, photographed and weighed. Tumors formed from NT control clone were significantly larger than E5 (*P* = 0.036) and E6 (*P* = 0.008) stable clones. NT tumors were the largest among the three experimental groups while E6 stable clone developed the smallest tumors. This trend was also reflected from the weight of the tumors, where the average weight of tumors from NT control clone was significantly higher than E6 stable clone (*P* = 0.032) (**Fig. 4C**). This result demonstrated that suppression of ILK in HCC cells attenuated the ability of HCC cells to form tumors in nude mice.

### ILK overexpression enhanced HCC cell growth and motility

**Figure 5:**
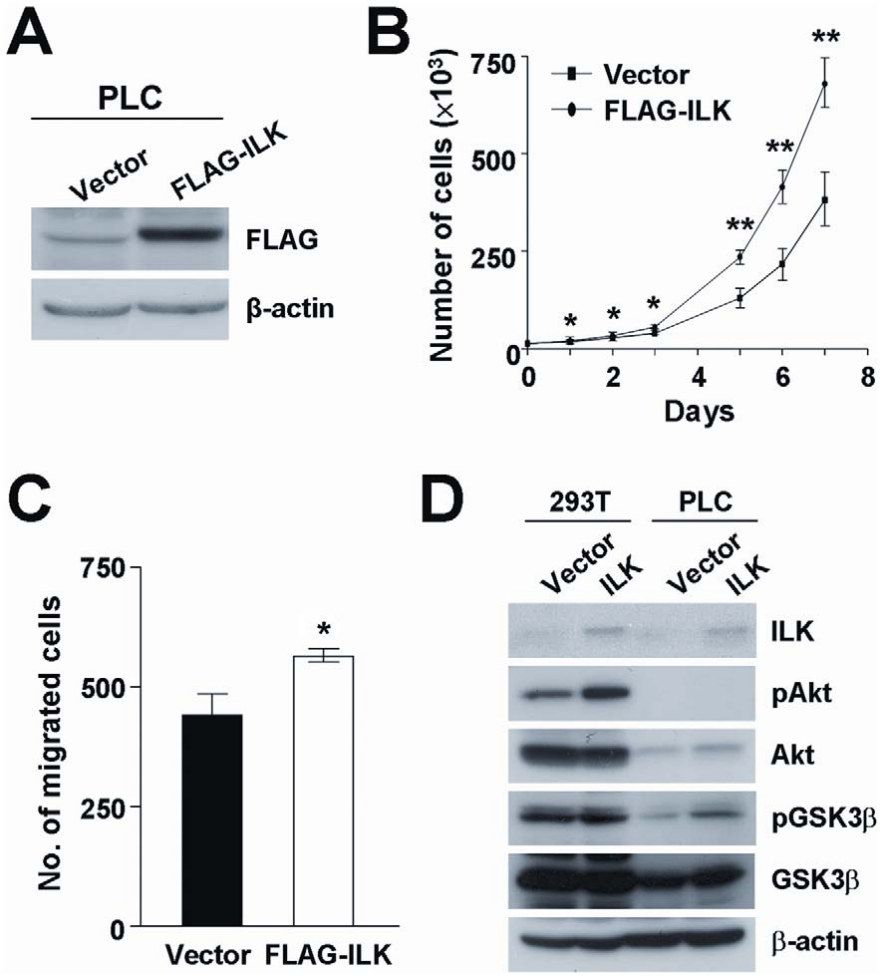
ILK overexpression enhanced PLC cell growth and motility. (**A**) FLAG-tagged ILK was transduced into PLC cells and stable clone of ILK was established. Western blot analysis confirmed stable FLAG-ILK expression in PLC cells but not in vector control clone. (**B**) PLC/vector and PLC/FLAG-ILK cells were counted in triplicates for 8 consecutive days. (**C**) PLC ILK overexpressing cells were subjected to migration assay. Cells were seeded in triplicates and allowed to migrate for 16 hours. Migrated cells were fixed and stained by crystal violet. (**D**) PLC and HEK293T cells overexpressing ILK were collected for western blot analysis to study the phosphorylation of Akt and GSK3β. Expression of β-actin was included as an internal loading control. *P<0.05 and **P<0.001 were regarded as statistically significant.

Apart from knockdown approach, overexpression of ILK in HCC cells was adopted as a complementary method to characterize the role of ILK in HCC. HCC cell line PLC was employed as the working model for overexpression approach due to its relatively low expression level of endogenous ILK (**Fig. 1C**). Stable PLC overexpressing FLAG-ILK clone and vector control clone were established (**Fig. 5A**). PLC ILK overexpressing cells were subjected to proliferation and motility assays. Results revealed that ILK overexpressing cells displayed significantly enhanced cell proliferation (*P*<0.001) and migration (*P* = 0.010) when compared with the vector control cells (**Fig. 5B and 5C**).

### ILK expression correlated with phosphorylation of Akt and GSK3β

**Figure 6:**
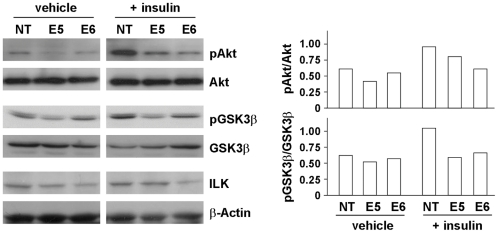
ILK knockdown suppressed phosphorylation of Akt and GSK3β. Insulin was added to cells to stimulate the PKB/Akt signaling pathway. Cell lysates were then collected for western blotting analysis. Expression levels of pAkt, total Akt, pGSK3β, total GSK3β and ILK were shown. Expression of β-actin was included as an internal loading control. The band intensities were determined by densitometry, and the amount of pAkt and pGSK3β was normalized with that of total Akt and GSK3β respectively.

Oncogenic Akt has been reported to be the main downstream player of ILK signaling pathways [7]. In the physiological context, it has been shown that ILK overexpression in HCC correlated with Akt activation [14]. This inspired us to investigate the involvement of Akt signaling in ILK-mediated oncogenic properties in HCC cells. BEL7402 ILK knockdown stable clones were treated with insulin to stimulate phosphorylation of Akt and its downstream effector GSK3β. In untreated cells, western blot analysis revealed higher pAkt Serine-473 and pGSK3β levels in NT control clone when compared to E5 and E6 stable clone. Upon insulin stimulation, the pAkt Serine-473 and pGSK3β levels were greatly elevated in NT control clone, but only increased marginally in E5 and E6 stable clones (**Fig. 6**). In accordance with the inhibition of Akt phosphorylation in ILK knockdown clones, pAkt Serine-473 expression level was also suppressed in tumors formed in subcutaneous injection (**Fig. 4D**). Conversely, overexpression of ILK in PLC cells enhanced phosphorylation of pGSK3β; however, pAkt Serine-473 was not even detected in the parental PLC cells. (**Fig. 5D**). Consistently, ILK overexpression induced upregulation of pAkt Serine-473 and pGSK3β levels in HEK293T cells. This implicated that ILK regulates the activity of Akt and which in turn exerts its functional effects in HCC cells.

## Discussion

Expression of ILK was found to be elevated in many human cancers. Correlation between ILK expression with tumor stage and patient survival has been reported in various malignancies including prostate cancer, colon cancer and ovarian cancer [10], [11], [12]. Nevertheless, research conducting on lymphoma, renal carcinoma and retinoblastoma revealed a decrease or no ILK expression in tumor tissues [15], [16]. Such heterogeneous expression pattern of ILK in human cancers may suggest tissue or organ-specific functions of ILK. Expression of ILK in human HCC has been examined and reported to be overexpressed [13], [14]. ILK overexpression was neither correlated with tumor grade nor patient survival. In this study, 57 cases of paired HCC and non-tumorous liver samples were subjected to qPCR analysis to assess the level of ILK mRNA. These cases were randomly selected which involved samples ranging from stage I to stage IV HCC. Here, we report that ILK was found to be overexpressed with at least two-fold in 37% of the cases. More important, a stepwise increase in ILK expression was revealed along tumor progression. ILK expression in either cirrhotic liver or stage I tumor was significantly lower than the advanced stage IV tumor.

In spite of the potential significance of ILK in hepatocarcinogenesis, functional role of ILK and elucidation of its associated pathways in HCC have not been clearly defined. Demonstration of its oncogenic activity in HCC is still lacking. To understand the functions of ILK, the endogenous ILK expression in two HCC cell lines was silenced by shRNA. Cell properties of the ILK-depleted cells were then analyzed and compared with the control cells in various functional assays. In both HCC cell lines, ILK knockdown stable clones displayed suppressed cell proliferation and anchorage independent growth. Besides, motility and invasiveness of cells were largely impeded upon ILK depletion. Our study has also provided the first validation about the functional loss of ILK expression *in vivo*. HCC cells with the suppressed ILK expression displayed inhibited ability to form tumors in nude mice. Notably, the results of functional assays indeed correlated very well with ILK knockdown efficiency. All these studies affirmed our findings that ILK exerts oncogenic effect on HCC cell lines and the extent of the effect depends highly on the degree of ILK expression in cells.

Phosphorylation of the pro-survival protein kinase B (PKB)/Akt at the serine 473 position in a phosphatidylinositol 3-kinase (PI3K)-dependent manner by ILK has been well established [7]. Persistent activation of Akt signaling cascade is prominent in various human cancers. Indeed, ILK/Akt pathway has been shown to be involved in breast cancer, prostate cancer and glioma [17], [18], [19]. Being the central player of signaling network, Akt is involved in diverse pathways and various physiological roles. Activation of the PKB/Akt pathway has been shown to induce cell survival, resist apoptosis, stimulate angiogenesis and enhance cell invasiveness of cancer cells [7], [20]. All these promoted growth and migratory capacities further facilitate tumor growth and progression. Due to the significance of PKB/Akt pathway on tumorigenesis and the well established link between ILK and Akt in other cancers, the involvement of Akt in ILK-mediated functional effect was initiated. Our result showed that the expression level of pAkt Serine-473 and pGSK3β were increased in NT control clone upon insulin stimulation, indicating that insulin could successfully activate Akt and GSK3β in BEL7402. On the contrary, the enhancement in pAkt Serine-473 and pGSK3β levels were not observed in the two ILK knockdown clones after insulin treatment. Our findings clearly indicate that silencing of ILK suppressed Akt phosphorylation and activation. On the other hand, enhanced level of pGSK3β was observed in PLC ILK overexpressing stable clone. Intriguingly, this implicated ILK-Akt signaling in HCC is supported by the previous findings in which ILK overexpression was strongly associated with the activation of Akt in HCC clinical tissues [14].

Akt is the key downstream component of PI3K signaling [21], [22]. Recruitment of Akt from the cytoplasm to the plasma membrane allows activation of Akt by phosphorylation of both Thr308 and Ser473 [23]. Activated Akt has been shown to phosphorylate and regulates a number of cellular proteins. Glycogen-synthase kinase 3 was identified as the first substrate of Akt followed by the subsequent identification of a number of targets phosphorylated by Akt [24], [25]. These substrates have been involved in various cellular processes such as metabolism, proliferation, differentiation, apoptosis, migration and invasion [26], [27]. Some downstream effectors of Akt have been shown to play an indispensible role in HCC. For instance, PAK1 expression was found to be elevated in about 75% of human HCCs [28]. Its overexpression was associated with more aggressive tumor behavior and poor prognosis. PAK1 overexpression and knockdown resulted in enhance and inhibited cell motility and invasiveness, respectively. Interestingly, study has shown that PAK1 phosphorylates ILK and mediates its nucleocytoplasmic shuttling and functions in the nucleus [29]. Another target of Akt is RhoA, which has also been implicated in HCC. RhoA is a member of the Ras superfamily of small GTPase. It plays important role in the regulation of actin cytoskeleton and stress fiber formation, as well as proper organization of focal adhesion complexes. As a result, activation of RhoA is important for cell migration and cell cycle progression [30]. Immunoblotting analysis revealed a higher RhoA expression in 69.2% (18/26) of HCC tumor tissues when compared with their adjacent non-tumorous tissues. Active RhoA level was also elevated in HCC tissues expressing high level of RhoA [31]. Higher expression of RhoA in HCC tumors was found to have significant association with the presence of venous invasion, microscopic satellite lesions, and advanced pTNM stage [32]. Kakinuma et al. has shown that constitutively active Akt increased the level of activated RhoA, where cells pre-treated with LY294002, a PI3K inhibitor, showed a dramatic decrease in active RhoA level [33]. Collectively, these studies show that ILK is tightly associated with important molecules which have been implicated in HCC tumourigenesis and motility. It is possible that ILK-Akt signaling with these important players might constitute important regulatory pathways in HCC.

## Materials and Methods

### Cell culture, HCC cell lines and clinical samples

HCC cell lines SMMC7721 and BEL7402 were obtained from the Shanghai Institute of Cell Biology, Chinese Academy of Sciences, People's Republic of China. Other human cell lines including HEK293FT, HepG2, Huh7, Hep3B and PLC were purchased from the American Type Culture Collection. H2P and MIHA cell lines were generous gifts from XY Guan and ST Fan of The University of Hong Kong respectively. Another HCC cell line, HLE was obtained from the Japanese Collection of Research Bioresources (JCRB, Japan) and was cultured in Dulbecco's Modified Eagle's Medium (DMEM) low glucose medium supplemented with 10% fetal bovine serum (FBS), penicillin and streptomycin. HEK293FT cells were maintained in DMEM high glucose medium and all the other cell lines were cultured in high glucose DMEM supplemented with sodium pyruvate. All cell lines were cultured in a humidified incubator at 37°C with 5% CO_2_ in air.

Human HCC tumor samples and their non-tumorous counterparts were obtained from HCC patients having surgical resection in Queen Mary Hospital. The clinicopathological parameters of HCC samples are shown in Table 1. The resected tissues were immediately snap-frozen in liquid nitrogen and stored at −80°C. Written informed consent from patients and approval of the human specimens used in this study was obtained by the Institutional Review Board of The University of Hong Kong/Hospital Authority Hong Kong West Cluster (HKU/HA HKW IRB).

**Table 1:** Summary of the clinicopathological characteristics of HCC patients.

Parameters	No. of patients (%)
AgeMean (range)SexMaleFemale	54.2 (28–82 years)45 (85)8 (15)
Venous invasionAbsentPresent	26 (51)25 (49)
Tumor encapsulationAbsentPresent	29 (57)22 (43)
Tumor microsatelliteAbsentPresent	28 (55)23 (45)
Hepatitis B surface antigen statusNegativePositive	11 (22)40 (78)
Resection marginNegativePositive	47 (92)4 (8)
Direct liver invasionAbsentPresent	28 (58)20 (42)
Cellular differentiation(Edmondson's grading)I-IIIII-IV	28 (55)23 (45)
Tumor size<5 cm≥5 cm	18 (35)33 (65)
Non-tumorous liver statusNormal and chronic hepatitisCirrhosis	22 (43)29 (57)
Number of tumor nodule1≥2	46 (90)5 (10)

### Protein extraction and western blot analysis

Proteins were extracted from cells using NET-N lysis buffer (25 mM Tris-HCl, pH 8.0, 50 mM NaCl, 0.2 mM EDTA, 0.1% NP40) with the addition of a cocktail of protease inhibitor (Roche, Mannheim, Germany). The cell lysate was cleared by centrifugation and the amount of protein was determined by Bradford method (Bio-Rad, Hercules, CA, USA). Proteins were subjected to electrophoresis and transferred to protein membrane (Amersham, Little Chalfont, UK). Immunodetection was performed by the ECL™ detection system (GE Healthcare, Freiburg, Germany) according to manufacturer's instructions. The following antibodies were used for western blotting. Anti-ILK1, anti-pAkt-Ser473, anti-Akt and anti-pGSK3β antibodies were from Cell Signaling Technology. Anti-GSK3β antibody was purchased from BD Bioscience and anti-β-actin antibody was from Sigma-Aldrich. The band intensity in the western blot was measured by AlphaEase FC Software (Alpha Innotech Corporation, San Leandro, CA).

### Quantitative real-time RT-PCR

Taqman® Gene Expression Assay (Applied Biosystems) specific for ILK was used to detect the expression level of ILK in HCC tumor samples using quantitative real time PCR (Assay ID: Hs00177914_ml). A Taqman® pre-developed assay reagent (Applied Biosystems) was used to determine the expression level of a housekeeping gene hypoxanthine ribosyltransferase (HPRT) (Assay ID:Hs99999909_ml), which was used for normalization. The quantitative PCR was performed according to manufacturer's manual and described elsewhere [34].

### ILK knockdown and overexpression stable clones establishment by lentiviral-based system

MISSION® Lentiviral shRNA knockdown system (Sigma-Adrich, St. Louis, MO) was used to knockdown ILK expression (NM_004517) in BEL7402 and HLE cells. Four shILK sequences were designed, namely E5, E6, E7 and E8. Preparation of viral supernatant was prepared according to manufacturer's instructions. In brief, shILK lentiviral vectors which pre-mixed with MISSION® lentiviral packaging mix (Sigma-Aldrich, St. Louis, MO) were transfected into 293FT cells using FuGENE® 6 transfection reagent (Roche Diagnostics GmbH, Germany). Twenty-four hours after transfection, viral particles containing the shILK sequence were rapidly produced and released to the culture medium. The medium containing viral particles were collected for infection or stored at −80°C for future use. For viral infection of HCC cells, viral supernatant was added to the cells. Polybrene was added to enhance the infection efficiency. Twenty-four hours after infection, the viral supernatant was removed and replaced with full medium. Cells were then cultured in puromycin (Calbiochem) containing medium to select for the positively-infected cells. To establish ILK overexpressing stable clone in PLC cells, pLenti6/V5-D-TOPO plasmid carrying FLAG-ILK and MISSION® Lentiviral Packaging mix were transfected into 293FT cells. Viral supernatant was collected and used to induce PLC cells. Transduced PLC cells were then selected by blasticidin (Invitrogen).

### Cell proliferation assay

To assess cell proliferation of each cell line, a growth curve was determined by seeding 10,000 cells on each well of a 12-well tissue culture plate and counting the number of cells in triplicate for five to eight consecutive days.

### Soft agar colony formation assay

Bottom agar (1 g agar, 30 of ml water, 50 ml of 2× DMEM, 20 ml of FBS) was equilibrated in a 55°C water bath while top agar (0.2 g agar, 15 ml of water, 25 ml of 2× DMEM, 10 ml of FBS) was put in a 45°C water bath. Five ml of bottom agar was added to each 60-mm tissue culture plate and was allowed to set at room temperature. A cell suspension of 8×10^4^ cells in 8 ml of top agar was prepared and 2 ml of it was added per 60-mm tissue culture plate pre-coated with 5 ml of bottom agar. After the agar was set, the plates were wrapped with parafilm and incubated at 37°C for three to four weeks to allow the formation of colonies. At the end of incubation, pictures of colonies formed in each plate were taken at three different views. Number of colonies and the size of them were then counted and measured.

### Wound-healing assay

HCC cells were seeded onto 6-well tissue culture plate until reaching 100% confluence. Mitomycin C (Calbiochem) at a concentration of 10 µg/ml was used to treat cells for 3 hours to inhibit cell proliferation. A wound was created by scratching the cell surface with a 10 µl pipette tip. Images of the wound were captured every 16, 24, 48 and 72 hours to observe the rate of wound closure.

### Cell migration and invasion assay

Cells were pretreated with mitomycin C (Calbiochem) for 3 hours to inhibit cell proliferation before subjected to migration and invasion assays. Transwell® (Corning) chamber was used to perform cell migration assay. To each chamber, 5×10^4^ cells were seeded and full medium was added to the bottom chamber as a chemo-attractant to the cells. For invasion assay, 5×10^5^ cells were seeded to each BD BioCoat™ Matrigel™ Invasion Chamber (BD Biosciences). For both assays, cells were allowed to migrate or invade. After incubation, cells on the upper side of the membrane were removed by cotton swab, while those at the bottom were fixed and stained with crystal violet. Numbers of cells migrated and invaded were counted. The assays were performed in triplicates. The detailed procedure has been described in previous studies [35].

### Nude mice subcutaneous injection

A cell suspension of 100 µl of 1× PBS containing 5×10^6^ cells was injected subcutaneously into the right flank of nude mice. Each experimental group was tested in a group of 5 mice. Size of the tumor formed was monitored closely and was measured every week using a caliper. Volumes of tumors were estimated according the formula: *Volume  = ½×a×b^2^*, where (*a*) and (*b*) represented the largest and smallest diameters respectively. Mice were sacrificed when the tumor size was larger than 1 cm×1 cm. Tumors were harvested, weighed and photographed. The animal work was performed according to the Animals (Control of Experiments) Ordinance (Hong Kong) and followed the University's guidelines on animal experimentation. The research protocol (CULATR 1749-08) was approved by the Committee on the Use of Live Animals in Teaching and Research (CULATR) of the University.

### Statistical analysis

ILK expression level in tumor and non-tumorous liver tissues and the expression of ILK in different stages of HCC tumors were compared using the paired and unpaired *t*-test, respectively. Student *t*-test was used as the statistical method to determine the difference in result distribution among cell groups in functional assays. The *t*-test could only compare a pair of cell lines at a time. All the above statistical analyses were performed by GraphPad Prism 3.00 (San Diego, California, USA). A *P*-value less than 0.05 is considered as statistically significant.
